# Antioxidant, anti-inflammatory and anti-septic potential of phenolic acids and flavonoid fractions isolated from *Lolium multiflorum*

**DOI:** 10.1080/13880209.2016.1266673

**Published:** 2016-12-09

**Authors:** Ki-Choon Choi, Young-Ok Son, Jung-Min Hwang, Beom-Tae Kim, Minseon Chae, Jeong-Chae Lee

**Affiliations:** a Grassland and Forages Research Center, National Institute of Animal Science, Cheonan, South Korea;; b Cell Dynamics Research Center and School of Life Sciences, Gwangju Institute of Science and Technology, Gwangju, South Korea;; c Research Center of Bioactive Materials, Chonbuk National University, Jeonju, South Korea;; d Institute of Oral Biosciences and School of Dentistry, Chonbuk National University, Jeonju, South Korea

**Keywords:** Italian ryegrass, lipopolysaccharide, reactive oxygen species, sepsis, bioactive compounds

## Abstract

**Context:** Interest has recently renewed in using *Lolium multiflorum* Lam. (Poaceae) (called Italian ryegrass; IRG) silage as an antioxidant and anti-inflammatory diet.

**Objective:** This study investigated the antioxidant, anti-inflammatory and anti-septic potential of IRG silage and identified the primary components in IRG active fractions.

**Materials and methods:** Total 16 fractions were separated from the chloroform-soluble extract of IRG aerial part using Sephadex LH-20 column before HPLC analysis. Antioxidant and anti-inflammatory activities of the fractions at doses of 0–100 μg/mL were investigated using various cell-free and cell-mediated assay systems. To explore anti-septic effect of IRG fractions, female ICR and BALB/c mice orally received 40 mg/kg of phenolic acid and flavonoid-rich active fractions F_7_ and F_8_ every other day for 10 days, respectively, followed by LPS challenge.

**Results:** The active fractions showed greater antioxidant and anti-inflammatory potential compared with other fractions. IC_50_ values of F_7_ and F_8_ to reduce LPS-stimulated NO and TNF-α production were around 15 and 30 μg/mL, respectively. Comparison of retention times with authentic compounds through HPLC analysis revealed the presence of caffeic acid, ferulic acid, myricetin and kaempferol in the fractions as primary components. These fractions inhibited LPS-stimulated MAPK and NF-κB activation. Supplementation with F_7_ or F_8_ improved the survival rates of mice to 70 and 60%, respectively, in LPS-injected mice and reduced near completely serum TNF-α and IL-6 levels.

**Discussion and conclusion:** This study highlights antioxidant, anti-inflammatory and anti-septic activities of IRG active fractions, eventually suggesting their usefulness in preventing oxidative damage and inflammatory disorders.

## Introduction

Diet is known to be an important factor in maintaining a healthy life. Increased interest has focused on the application of dietary components to prevent inflammatory damage and/or to control immune response induction (Kamiyama & Shibamoto 2012; Yamaura et al. 2012; Sun et al. 2016). Vitamins and phenolic compounds are bioactive antioxidants that are capable of preventing oxidative damage and degenerative disorders (Jomova & Valko 2011; Ashraf et al. 2016; Bouriche et al. 2016). Accordingly, diets abundant in antioxidants and anti-inflammatory constituents are believed to be an effective means of improving the resistance to pathogens.

As preventive and therapeutic approaches addressing inflammatory diseases gain importance, numerous investigators have focused their efforts on the identification of anti-inflammatory substances from natural materials. Antioxidant phytochemicals, anti-inflammatory agents and functional nutrients are more abundant in plants than grains (Quinde-Axtell & Baik 2006; Zilić et al. 2012). Plants are in fact valuable resources for a wide range of secondary metabolites having medicinal and pharmaceutical activities (Rao & Ravishankarb 2002; Erel et al. 2011). Interestingly, the feeding of fresh forage (either as pasture plus a concentrate or as a silage-based total mixed ration) is believed to improve the quality and quantity of animal products rather than the concentrate-rich diets did. It was also reported that the feeding of fresh forage increased animal health, even for monogastric animals (Cuevas Montilla et al. 2011; Firuzi et al. 2011). These beneficial effects of plant-based diets are considered to be related to the presence of bioactive metabolites such as phenolic acids and flavonoidic compounds.

*Lolium multiflorum* Lam. (Poaceae) (also called Italian ryegrass; IRG) is widely cultivated in the world for various purposes. IRG is known to contain highly active and nutritional compounds (Ilavenil et al. 2014). We previously confirmed the presence of α-linolenic acid, docosahexaenoic acid, oleic acid, docosatetraenoic acid and caprylic acid in IRG silage and identified these fatty acids as the major compounds responsible for IRG-stimulated adipogenesis (Valan Arasu et al. 2014). The chloroform extract of IRG silage also exhibited greater scavenging potential on reactive oxygen species (ROS), compared with the extracts of sorghum, barley and alfalfa (Hwang et al. 2013). In addition, the IRG chloroform extract showed 85, 75 and 83% suppression on the production of nitric oxide (NO), tumour-necrosis factor-α (TNF-α) and interleukin-6 (IL-6), respectively, in lipopolysaccharide (LPS)-stimulated macrophages (Hwang et al. 2013). These antioxidant and anti-inflammatory effects of IRG extract were closely correlated with the content of polyphenolic compounds. Taken as a whole, we suggested that IRG silage could potentially be the attractive source as a functional diet. However, little is known regarding the compounds responsible for its antioxidant and anti-inflammatory activities. The mechanisms by which IRG silage extract exerts its biological activity are not investigated.

In this study, we initially separated total 16 subfractions (F_1_ to F_16_) from the chloroform-soluble extract of IRG silage methanol extract. Based on the antioxidant and anti-inflammatory activities, we selected the two fractions F_7_ and F_8_ as IRG active fractions and identified the primary constituents in the fractions through high-performance liquid chromatography (HPLC) analysis. We also investigated the mechanisms by which the active fractions exert antioxidant and anti-inflammatory potential using *in vitro* and *in vivo* assay systems.

## Materials and methods

### Chemicals

Dulbecco’s modified Eagle’s medium (DMEM), foetal bovine serum (FBS) and phosphate-buffered saline (PBS) were purchased from Gibco (Gaithersburg, MD). Monoclonal antibodies specific for cyclooxygenase 2 (COX-2), inducible nitric oxide synthase (iNOS) and β-actin were obtained from Santa Cruz Biotechnology (Santa Cruz, CA). Authentic compounds such as (+)-catechin, caffeic acid, ferulic acid, *p*-coumaric acid, syringic aldehyde, myricetin, propyl gallate, quercetin and kaempferol were purchased from Sigma-Aldrich Co. LLC (St. Louis, IL). Unless otherwise specified, other chemicals were purchased from Sigma Chemical Co. and all plastics were Falcon Labware (Becton-Dickinson, Franklin Lakes, NJ).

### Mice and ethics statement

Female BALB/c and ICR mice (≥ 20 g; Damul Science Co., Dajeon, South Korea) were housed in automatically controlled conditions at 22 ± 1 °C with 12 h light/dark cycle and 45–55% relative humidity. All mice had free access to standard rodent food pellets and water. This study was carried out in strict accordance with the recommendations in the Guide for the Animal Care and Use of the Chonbuk National University (Jeonju, South Korea). The protocol was approved by the University Committee on Ethics in the Care and Use of Laboratory Animals (Permit No. CBU 2012-0039). Due to the experimental intervention, animals died without euthanasia during the experimental periods. Specifically, the protocol describing the reason of animal death without euthanasia and the mortality aspects was reviewed and approved by the animal ethics committee of the University. The health of the animals was monitored every 6 h per day during the experimental periods. The animals living at the end of experiments were euthanized according to the guidelines of the animal ethics committee.

### Extraction and fractionation of IRG extract

The aerial parts of IRG were collected in September 2011 from the Grassland and Forages Research Center at the National Institute of Animal Science (NIAS) (Cheonan, South Korea). The IRG sample was authenticated by a taxonomist at NIAS, harvested at the flowering stage, and ensiled. IRG silages were collected at 40 days after cultivation, dried at 60 °C for three days, and pulverized in an electric grinder to yield a coarse powder. Initially, crude methanol extract was obtained from 771 g of IRG powder, concentrated using a rotary vacuum evaporator (Rotavapor R110, Büchi, Switzerland), and lyophilized as described previously (Choi et al. 2013; Hwang et al. 2013). The IRG methanol extract was serially extracted with the following solvents in a stepwise manner: petroleum ether, chloroform, *n*-butanol and distilled water. Based on the ability to inhibit NO production in LPS-stimulated macrophages, the IRG chloroform-soluble extract was further separated into 16 subfractions (F_1_ to F_16_) by chromatography using a Sephadex LH-20 column (Supplemental Figure 1). Briefly, 100 g of Sephadex LH-20 resin (LH20100-100G) was hydrated in deionized water overnight at 4 °C and packed into a glass column (370 × 30 mm). The IRG chloroform extract (3.936 g) was loaded onto the top of an LH-20 cartridge and sequentially eluted with H_2_O (1/3 of the bed volume) to obtain one fraction (F_1_). Then, the chloroform extract was serially fractionated with 2 × (v/v) of the bed volume of 10, 30, 50, 70 and 100% methanol, thereby yielding 15 fractions (F_2_ to F_16_; three fractions for each methanol gradient elution). The organic solvent of each fraction was evaporated and the remaining extracts were lyophilized to a powder.

### HPLC conditions

The components of IRG fractions were identified by reversed-phase HPLC. All samples were filtered through a 0.45 μm pore syringe-driven filter before injection. Briefly, 10 μL of each sample was resolved using a Sykam HPLC system equipped with a S 2100 solvent-delivery system (Sykam, Gewerbering, Germany) and a S 7131 Reagent Organizer (Sykam) and then separated through an 18-ODS Hector C18-M51002546 column (250 mm ×4.6 mm, 5 μm) (RStech Co., Ltd. Daejeon, South Korea). The mobile phase consisted of purified water with 0.1% formic acid (solvent A) and acetonitrile (solvent B) at a flow rate of 1.0 mL/min. Gradient elution was performed as follows: 0 to 1 min, 10% solvent B; 1 to 20 min, linear gradient from 10 to 100% solvent B; 20 to 21 min, 100% solvent B; 21 to 26 min, linear gradient from 100 to 10% solvent B; and 26 to 33 min, 10% solvent B. Authentic (reference) compounds and IRG fractions were detected at a wavelength of 280 nm. Phenolic compounds in the IRG fractions were identified by comparing their relative retention times with those of the authentic compounds.

### Deoxyribose assay

The nonsite-specific scavenging activity of IRG fractions on hydroxyl radicals was assessed using a deoxyribose assay method as described previously (Halliwell et al. 1987). Briefly, 100 μg of each fraction was mixed with 0.5 mL reaction buffer (100 μM FeCl_3_, 104 μM EDTA, 1.5 mM H_2_O_2_, 2.5 mM deoxyribose and 100 μM l-ascorbic acid, pH 7.4) and incubated for 1 h at 37 °C. Next, 1 mL of 0.5% 2-thiobarbituric acid in 0.025 M NaOH and 0.5 mL of 2.8% trichloroacetic acid were added into the mixture followed by incubation for 30 min at 80 °C. The absorbance of the resultant product was measured at 532 nm using a Beckman DU^®^ 530 spectrophotometer (GMI, Ramsey, MN).

### Superoxide radical scavenging assay

The superoxide radical scavenging activity of each IRG fraction was determined by xanthine/xanthine oxidase assay with a slight modification (Gotoh & Niki 1992). Briefly, 100 μg of each fraction was mixed with a buffer containing 50 μL of 30 mM EDTA (pH 7.4), 20 μL of 20 mM hypoxanthine in 50 mM NaOH and 100 μL of 1.42 mM nitro blue tetrazolium (NBT) in a tube. After preincubation at room temperature for 5 min, 100 μL of 0.25 U/mL xanthine oxidase was added to the mixture and the volume was brought up to 1 mL with 50 mM phosphate buffer (pH 7.4). After incubation for 20 min, the absorbance of the reaction product was measured at 560 nm.

### DPPH radical scavenging assay

The 2,2-diphenyl-1-picrylhydrazyl (DPPH) radical-scavenging activity of each IRG fraction was also assessed as previously described (Baek et al. 2011). Initially, 100 μg of each fraction was mixed with 1 mL of 0.1 mM DPPH-ethanol solution and 450 μL of 50 mM Tris-HCl buffer (pH 7.4) and then incubated for 30 min at room temperature. The reduction of DPPH radicals was measured by reading the absorbance of the reaction product at 517 nm.

### Electron spin resonance (ESR) assay

All ESR measurements were conducted using a Bruker EMX spectrometer (Bruker Instruments, Billerica, MA) and a flat cell assembly as described previously (Yu et al. 2011). In brief, 5,5-dimethyl-1-pyrroline-1-oxide was charcoal-purified and distilled to remove ESR-detectable impurities prior to use. Data acquisition and analysis were performed with Acquisit (Bruker Instruments). Each reaction was set up in a test tube in a final volume of 0.5 mL. The reaction mixture was then transferred to a flat cell for ESR measurement. Experiments were performed at room temperature under ambient air.

### Cell culture and treatments

RAW 264.7 macrophages were cultured in DMEM supplemented with 10% FBS, 2 mM l-glutamine, and antibiotics. Cells (1 × 10^6^ cells/mL) were seeded in 2 mL or 200 μL medium in 6-well or 96-well flat-bottomed plates, respectively. After reaching 70–80% confluence, cells were exposed to various concentrations (0–100 μg/mL) of the IRG fractions or each of several phenolic compounds, both in the presence and absence of 1 μg/mL LPS. At various times of incubation, culture supernatants or cells were harvested and processed for further analyses.

### Anti-inflammatory activity assays

The anti-inflammatory activity of IRG fractions was determined by measuring the levels of NO, IL-6 and TNF-α in LPS-stimulated macrophages. In brief, cells were incubated with IRG fractions before exposure to LPS for 48 h and culture supernatants were then collected. Nitrite concentration in the culture medium was measured as an indicator of NO production using the Griess reagent (Fang et al. 2011). The levels of IL-6 and TNF-α in the culture supernatants were determined using mouse Quantikine^®^ ELISA kits (R&D Systems, Minneapolis, MN) according to the manufacturer’s instructions.

### Western blot analysis

Whole protein lysates were extracted by resuspending cells in a lysis buffer as described previously (Son et al. 2009) and the protein content in each sample was quantified according to the Bradford method (Bradford 1976). The protein extracts (20 μg/sample) were separated by SDS–PAGE on 12% gels and electroblotted onto PVDF membranes. The membranes were sequentially probed with primary and secondary antibodies prior to treatment with enhanced chemiluminescence (Amersham Pharmacia Biotech, Buckinghamshire, UK). Finally, the immunoreactive bands were visualized by exposing the blots to X-ray film (Eastman-Kodak, Rochester, NY).

### Enzymatic mitogen-activated protein kinase (MAPK) activity assay

The levels of extracellular signal-regulated kinase (ERK) and p38 MAPK activity were determined using immunometric assay kits according to the manufacturer’s instructions. Briefly, RAW264.7 cells were exposed to 1 μg/mL LPS in the presence or absence of IRG active fractions for 1 h. Cell lysates were prepared in RIPA buffer (50 mM Tris-Cl, pH 7.4, 150 mM NaCl, 1 mM EDTA, 1 mM EGTA, 1% Triton X-100, 1% sodium deoxycholate and 0.1% SDS). Equal amounts of proteins were spotted onto the microtitre plates of a p-ERK or p-p38 MAPK enzyme assay kit (Assay Designs Inc., Ann Arbor, MI). The resultant absorbance was measured at 450 nm.

### Assays for NF-κB DNA-binding activity

The binding activity of NF-κB to its specific DNA sequence was determined by an electrophoretic mobility shift assay (EMSA). To this end, RAW 264.7 macrophages were stimulated with 1 μg/mL LPS for 1 h in the presence and absence of IRG active fractions (0–100 μg/mL per fraction). Nuclear protein extracts were prepared as previously described (Son et al. 2009) and DNA-protein binding reactions were performed for 30 min according to the methods described elsewhere (Choi et al. 2013). DNA-protein complexes were separated on 6% polyacrylamide gels and the gels were dried followed by exposure to X-ray film (Eastman Kodak Co.) for 12 to 24 h at −70 °C. The oligonucleotide sequences specific for NF-κB were as follows: 5′-AAG GCC TGT GCT CCG GGA CTT TCC CTG GCC TGG A-3′ and 3′-GGA CAC GAG GCC CTG AAA GGG ACC GGA CCT GGA A-5′. DNA binding activity of the NF-κB p65 subunit was also examined by determining peroxidase activity of the nuclear protein extracts. This assay was carried out using a TransAM^®^ NF-κB p65 ELISA kit (Active Motif, Carlsbad, CA) according to the manufacturer’s instructions. The resultant binding activity of the p65 subunit was evaluated by measuring the absorbance of each reaction at 450 nm using a SpectraCount^TM^ ELISA reader (Packard Instrument Co., Downers Grove, IL). In addition, the level of nuclear p65 was determined by Western blot analysis of the same nuclear extracts.

### Assays for survival rate and serum cytokine levels

Female ICR or BALB/c mice were randomly divided into three groups (*n* = 15/group) and caged separately (five mice/cage) before treatment with LPS and/or IRG active fractions. Mice were orally administered with either 200 μL PBS alone or 200 μL PBS containing F_7_ or F_8_ (40 mg/kg body weight) every other day for 10 days. Mice received an intraperitoneal injection of LPS (30 mg/kg body weight) one day after the last administration. The survival rate in animal groups (*n* = 10/group) was recorded every 6 h after the LPS injection for four days. Animals lived at the end of experiments were sacrificed by CO_2_ asphyxiation. Serum levels of TNF-α and IL-6 were also measured in a murine BALB/c model of sepsis. Serum samples were collected by retro-orbital bleeding from five mice that had been injected with LPS alone or pretreated with an IRG fraction in each group. Serum samples were collected at 2 and 8 h after the LPS injections. The serum levels of TNF-α and IL-6 were measured using mouse Quantikine^®^ ELISA kits (R&D Systems). The mice used for serum collection were not included in the calculation of survival rates.

### Statistical analysis

Data are expressed as means ± standard deviation (SD). To identify significant differences among the groups, one-way analysis of variance (SPSS version 12.0 software, Chicago, IL) was performed, followed by Scheffe’s test. A *p* value <0.05 was considered significant.

## Results

### *In vitro* antioxidant and anti-inflammatory potential of IRG fractions

**Figure 1.**
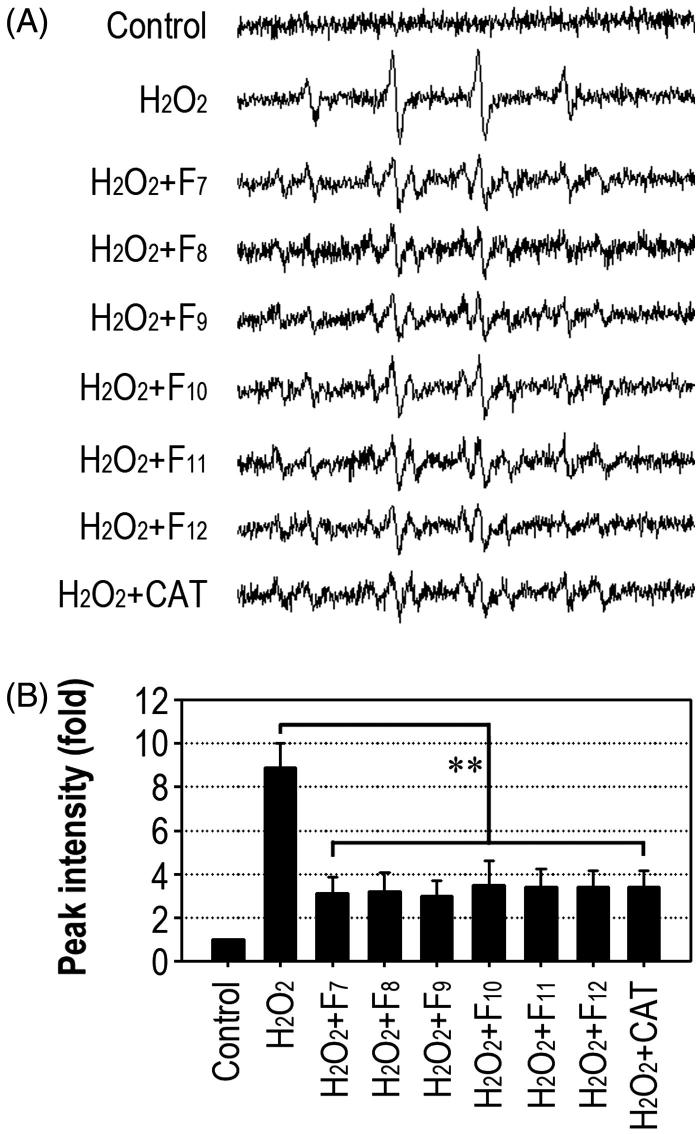
IRG fractions reduce intracellular ROS generation in macrophages. (A) Cells were exposed to 1 mM H_2_O_2_ in the presence of 1 mg/mL of each fraction or 500 U/mL CAT for 24 h. The resultant ESR spectra were recorded. (B) The fold changes in peak intensity were calculated from three different samples. **p < 0.01 vs. H_2_O_2_ treatment alone.

The fractions from F_7_ to F_12_ exhibited the strong inhibition of hydroxyl radical-induced deoxyribose degradation, in which F_7_ showed the most potent activity (Table 1). The fractions (F_7_ to F_12_) also suppressed superoxide anion radical-mediated reduction of NBT to a greater extent than other fractions, even though all the IRG fractions showed scavenging potential on DPPH radicals. We subsequently explored whether the IRG F_7_ to F_12_ fractions protect cells against H_2_O_2_-mediated oxidative damage by ESR assay (Figure 1). The H_2_O_2_-induced increase of ESR signal intensity in RAW 264.7 macrophages was significantly (*p* < 0.01) inhibited by adding the IRG fractions to the cultures (Figure 1(A,B)). Similarly, treatment with catalase (500 U/mL) reduced the signal intensity of the ESR spectrum (Figure 1(B)).

**Table 1.** Radical scavenging activities of the fractions from chloroform soluble extract^a^.

Fractions (100 μg/mL)	% Inhibition^b^		
	Hydroxyl radicals	Superoxide anion radicals	DPPH radicals
Fraction 1	13.2 ± 2.9	19.5 ± 3.5	33.4 ± 1.9
Fraction 2	29.1 ± 3.2	21.4 ± 2.1	32.6 ± 2.5
Fraction 3	16.1 ± 3.6	26.3 ± 3.8	29.4 ± 3.5
Fraction 4	18.4 ± 4.3	29.5 ± 3.2	38.1 ± 4.2
Fraction 5	17.2 ± 2.7	37.1 ± 3.7	32.5 ± 2.1
Fraction 6	21.2 ± 2.1	46.3 ± 4.1	42.6 ± 3.5
Fraction 7	74.3 ± 4.2	83.4 ± 3.8	87.2 ± 2.9
Fraction 8	71.3 ± 3.2	82.4 ± 3.9	79.3 ± 4.1
Fraction 9	66.3 ± 2.4	80.4 ± 4.1	86.1 ± 4.2
Fraction 10	70.2 ± 4.5	79.4 ± 3.2	78.1 ± 3.4
Fraction 11	59.3 ± 3.2	75.1 ± 3.8	67.9 ± 3.9
Fraction 12	62.1 ± 3.6	74.8 ± 3.7	68.6 ± 3.1
Fraction 13	23.7 ± 3.3	33.9 ± 3.1	43.1 ± 3.2
Fraction 14	44.9 ± 3.7	51.4 ± 4.2	48.3 ± 2.3
Fraction 15	51.3 ± 4.3	55.8 ± 3.4	61.2 ± 3.9
Fraction 16	18.1 ± 2.6	28.5 ± 3.1	38.4 ± 3.6

a Chloroform soluble extract of the IRG methanol extracts were processed for fractionation with Sephadex and LH20 column chromatography.

b The activities of fractions to scavenge free radicals directly and indirectly were determined (*n* = 3). The results were expressed as % inhibition, which was calculated as follows: % inhibition = [(OD_control_ − OD_sample_)/OD_control_] × 100.

Exposure of RAW 264.7 cells to 1 μg/mL LPS for 48 h resulted in a 10.3-fold increase in the nitrite concentration in culture medium, compared with the control value (Table 2). Fractions F_7_ to F_12_ significantly reduced the LPS-stimulated NO production in the cells, whereas the fractions F_1_, F_3_ to F_6_, F_13_ and F_16_ did not show any inhibitory effects. The fractions F_7_ to F_12_ also showed greater inhibitory activity against LPS-stimulated production of TNF-α and IL-6 than did other fractions. Considering the antioxidant and anti-inflammatory activities of these fractions, we finally selected F_7_ and F_8_ as the IRG active fractions and performed further experiments using the two fractions.

**Table 2.** Inhibitory effects of IRG fractions on LPS-induced NO and cytokine production^a^.

Fractions (100 μg/mL)	Nitrite (μM)	TNF-α (ng/mL)	IL-6 (ng/mL)
Control	5.1 ± 1.2	1.9 ± 0.8	0.2 ± 0.1
LPS alone^b^	52.3 ± 3.4	37.4 ± 4.3	8.7 ± 1.5
Fraction 1^c^	43.2 ± 2.9	33.2 ± 4.1	7.9 ± 2.2
Fraction 2	10.2 ± 5.9***	7.3 ± 2.5***	3.2 ± 1.7*
Fraction 3	43.1 ± 3.7	32.2 ± 4.8	7.5 ± 2.3
Fraction 4	35.1 ± 12.4	28.4 ± 6.3	7.4 ± 1.9
Fraction 5	38.6 ± 3.9	29.3 ± 3.4	7.7 ± 1.8
Fraction 6	41.2 ± 4.1	34.1 ± 5.9	6.9 ± 2.5
Fraction 7	5.3 ± 3.1***	2.5 ± 2.2***	1.1 ± 0.8**
Fraction 8	5.0 ± 0.8***	2.9 ± 1.3***	1.0 ± 0.9**
Fraction 9	6.9 ± 2.8***	2.6 ± 2.1***	1.0 ± 0.8**
Fraction 10	6.1 ± 2.2***	3.5 ± 3.6***	1.1 ± 1.0**
Fraction 11	5.8 ± 1.1***	2.8 ± 2.1***	2.8 ± 1.5*
Fraction 12	5.4 ± 1.8***	2.8 ± 2.4***	3.2 ± 0.9*
Fraction 13	32.6 ± 6.8	25.3 ± 3.9*	5.9 ± 2.2
Fraction 14	15.6 ± 9.2**	10.2 ± 5.1***	2.1 ± 1.8*
Fraction 15	11.2 ± 7.9**	8.4 ± 3.2***	2.9 ± 1.9*
Fraction 16	32.4 ± 12.6	27.2 ± 4.9	6.5 ± 2.4

a The ability of IRG fractions on anti-inflammatory action was determined by measuring their activity to inhibit LPS-mediated production of NO and pro-inflammatory cytokines (*n* = 3).

b Control cells were incubated without LPS and fractions.

c Cells were exposed to 1 μg/mL LPS without any IRG samples.

* *p* < 0.05.

** *p* < 0.01.

*** *p* < 0.001 vs. LPS treatment alone.

### Primary constituents of the IRG active fractions

**Figure 2.**
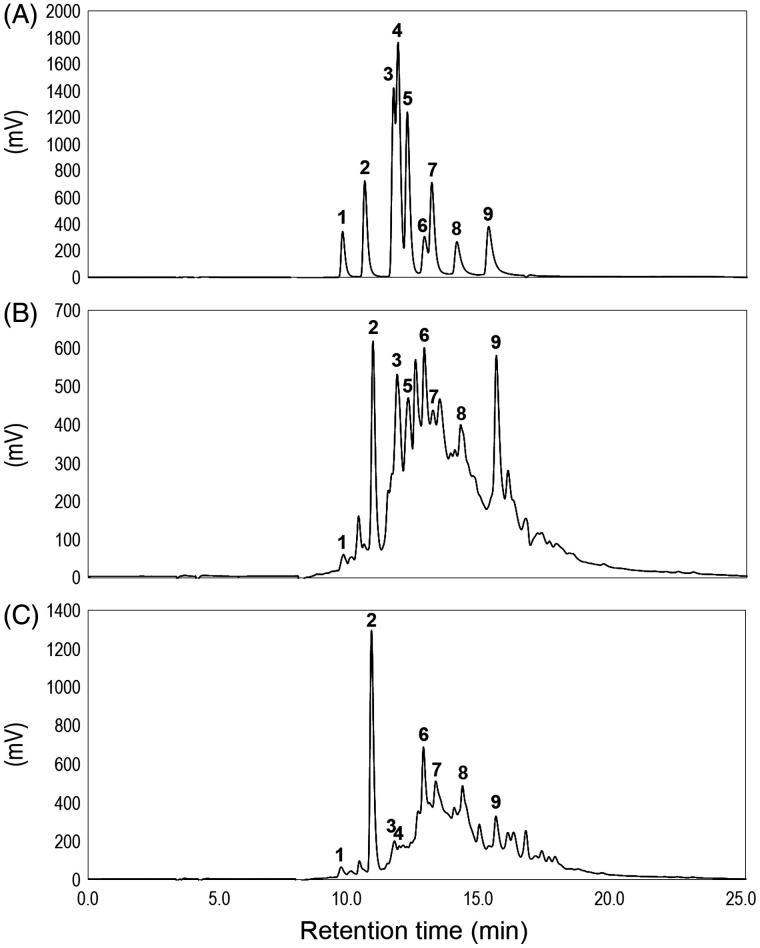
HPLC chromatograms of (A) the authentic compounds, (B) the IRG active fraction F_7_ and (C) the IRG active fraction F_8_. Peaks: 1, (+)-catechin; 2, caffeic acid; 3, ferulic acid; 4, *p*-coumaric acid; 5, syringic aldehyde; 6, myricetin; 7, propyl gallate; 8, quercetin; 9, kaempferol. Detection was performed at 280 nm.

We next determined the primary components consisted of F_7_ and F_8_ through HPLC analysis. The components in these fractions were identified by comparison of their retention times with those of authentic compounds. The HPLC chromatogram (recorded at 280 nm) of standards **1**–**9** [(+)-catechin (**1**), caffeic acid (**2**), ferulic acid (**3**), *p*-coumaric acid (**4**), syringic aldehyde (**5**), myricetin (**6**), propyl gallate (**7**), quercetin (**8**) and kaempferol (**9**)] is shown in Figure 2(A). The chemical structures and retention times of these compounds are described in Supplemental Figure 2. The authentic compounds used in this study were selected via a preliminary comparison of the recorded mass spectra with the standard mass spectra. HPLC chromatograms of F_7_ (Figure 2(B)) and F_8_ (Figure 2(C)) revealed similar component profiles with the different peaks depending on the elution solvent. The peak areas of compounds **2**, **3**, **6** and **9** in F_7_ were higher than those of the other compounds, whereas compound **4** was absent from this fraction. On the other hand, compound **2** was the major phenolic compound in F_8_, while compound **5** was not found. Compound **1** was detected at the lowest levels in both F_7_ and F_8_. HPLC analysis of other IRG fractions F_9_–F_12_ yielded quite different chromatograms from those of F_7_ and F_8_ (Supplemental Figure 3). These chromatograms also displayed many peaks that did not correspond to the authentic compounds.

### Anti-inflammatory activities of the IRG active fractions

**Figure 3.**
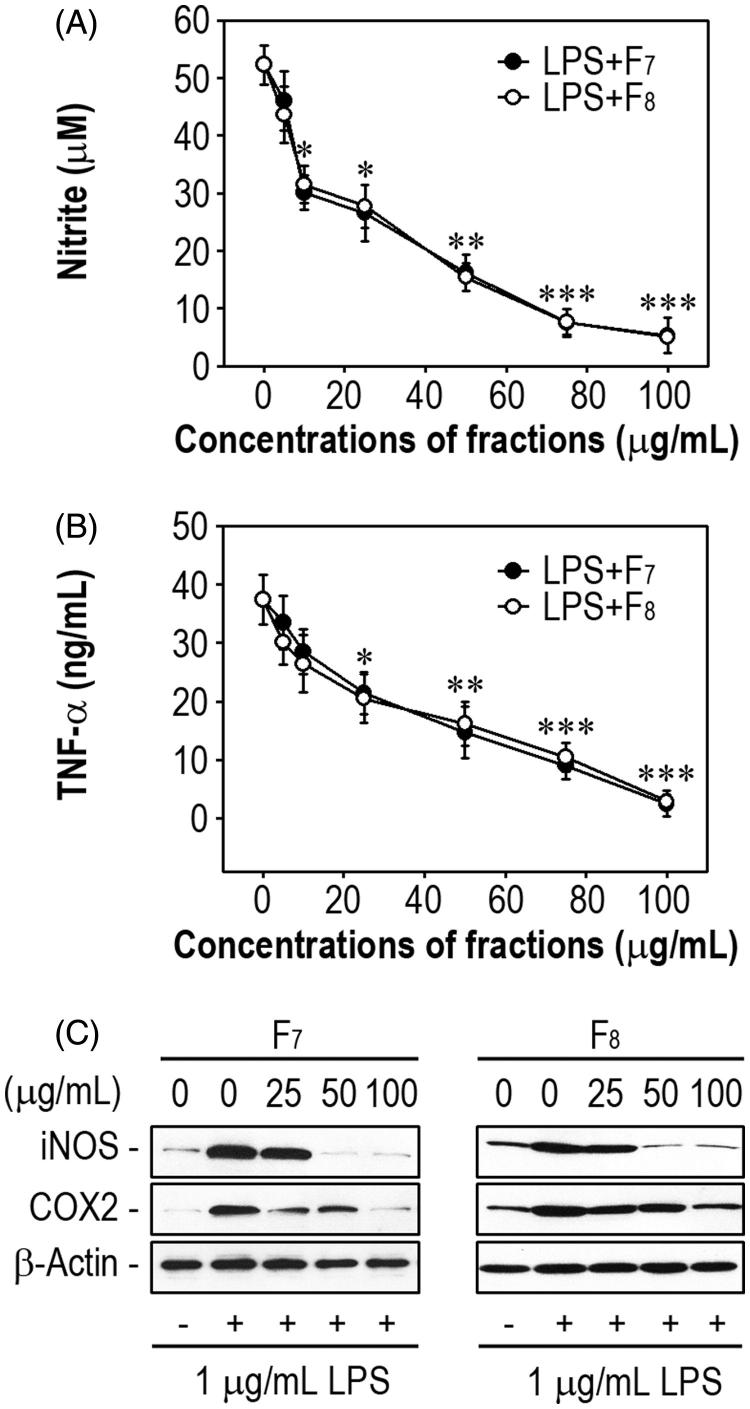
The IRG active fractions inhibit the production of NO and TNF-α and the induction of iNOS and COX-2 in LPS-stimulated RAW 264.7 macrophages. Cells were treated with various concentrations (0–100 μg/mL) of F_7_ or F_8_ prior to stimulation with 1 μg/mL LPS. After 48 h of incubation, the levels of (A) NO and (B) TNF-α in the conditioned media were determined. *p < 0.05, **p < 0.01, and ***p < 0.001 vs. LPS treatment alone. (C) Cells were treated with 100 μg/mL of each of the two active fractions in the presence of 1 μg/mL LPS for 24 h. Whole protein lysates were prepared from the cells and analyzed by Western blotting.

**Figure 4.**
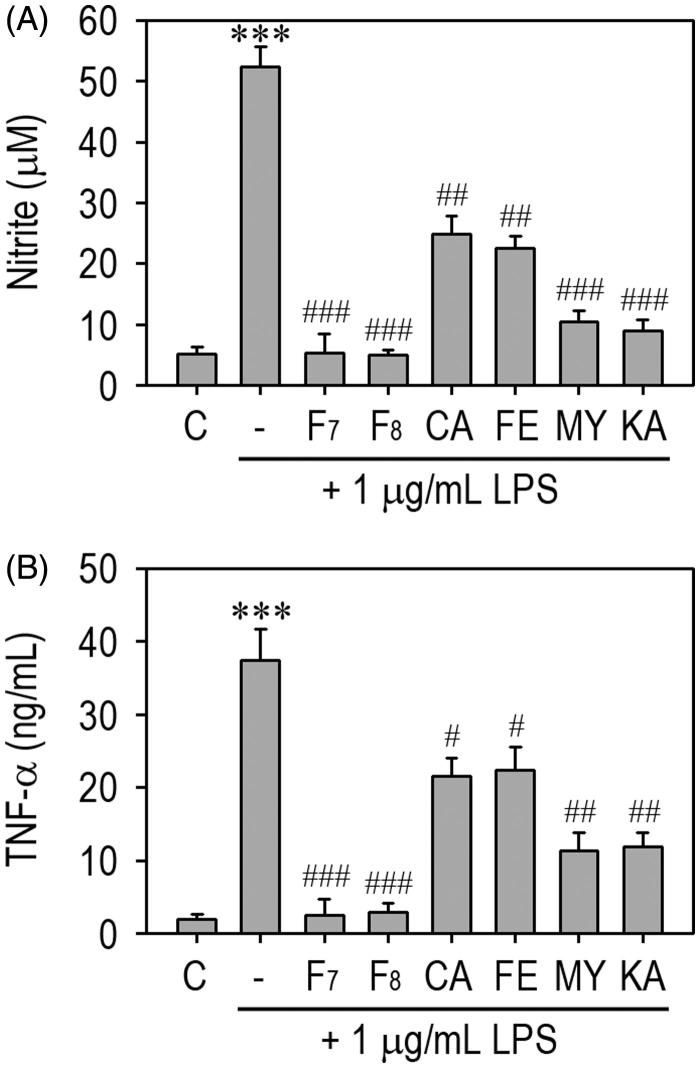
The IRG active fractions inhibit NO and TNF-α production in LPS-stimulated RAW 264.7 macrophages more than a single compound. Cells were treated with 100 μg/mL of F_7_, F_8_, caffeic acid (CA), ferulic acid (FA), myricetin (MY) or kaempferol (KA) prior to stimulation with 1 μg/mL LPS. After 48 h of incubation, the levels of (A) NO and (B) TNF-α in the conditioned media were determined. ***p < 0.001 vs. the untreated control values. #*p* < 0.05, ##*p* < 0.01, and ###*p* < 0.001 vs. LPS treatment alone.

We investigated the dose-dependent effects of F_7_ and F_8_ on LPS-stimulated production of inflammatory mediators in RAW 264.7 macrophages. These fractions inhibited LPS-mediated secretion of NO and TNF-α in the cells in a dose-dependent manner (Figure 3(A,B)). Concentration-activity curves revealed that the concentrations of F_7_ required to achieve 50% reductions in the levels of NO and TNF-α (IC_50s_) were approximately 15 and 30 μg/mL, respectively. Similar IC_50s_ were calculated for F_8_. Pretreatment with F_7_ or F_8_ (50 μg/mL) almost completely suppressed the induction of iNOS in LPS-stimulated macrophages. The addition of F_7_ also inhibited apparently LPS-stimulated COX-2 induction even at the concentration of 25 μg/mL, whereas F_8_ appeared to attenuate the induction only at 100 μg/mL. We subsequently compared the anti-inflammatory activities between the active fractions and four single compounds consisted mainly of the fractions. The fractions F_7_ and F_8_ (100 μg/mL) showed greater activities to inhibit LPS-stimulated production of NO (Figure 4(A)) and TNF-α (Figure 4(B)) than did each of single compounds such as caffeic acid, ferulic acid, myricetin and kaempferol at the same concentration.

### Anti-inflammatory mechanisms of the IRG active fractions

**Figure 5.**
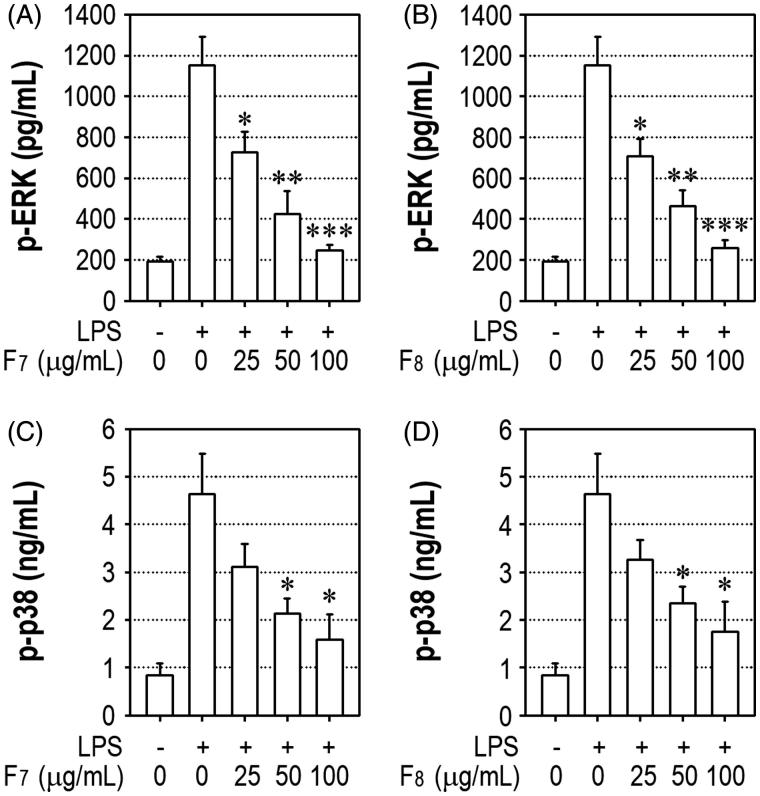
The IRG active fractions inhibit ERK and p38 MAPK phosphorylation in LPS-stimulated RAW 264.7 cells. Cells were stimulated with 1 μg/mL LPS in the presence or absence of F_7_ or F_8_ at the indicated concentrations. After 1 h of incubation, the cellular levels of (A and B) p-ERK and (C and D) p-p38 MAPK were measured by enzymatic assays. *p < 0.05, **p < 0.01, and ***p < 0.001 vs. LPS treatment alone.

Since ERK- and p38 MAPK-mediated signalling is critical for the induction of LPS-stimulated inflammatory responses, we assessed the inhibitory effects of F_7_ and F_8_ on LPS-stimulated increases in p-ERK and p-p38 levels. LPS stimulation resulted in a dramatic upregulation of p-ERK1/2 to 1,150 ± 140 pg/mL, which was much higher than the control level (193 ± 21 pg/mL) (Figure 5(A)). However, the addition of 50 and 100 μg/mL of F_7_ decreased p-ERK level to 425 ± 111 and 246 ± 28 pg/mL, respectively. Similarly, pretreatment with F_8_ attenuated the LPS-mediated increase in p-ERK (Figure 5(B)). LPS-mediated increase in p-p38 MAPK level was also significantly (*p*** **< 0.05) attenuated by pretreatment with either F_7_ (Figure 5(C)) or F_8_ (Figure 5(D)).

**Figure 6.**
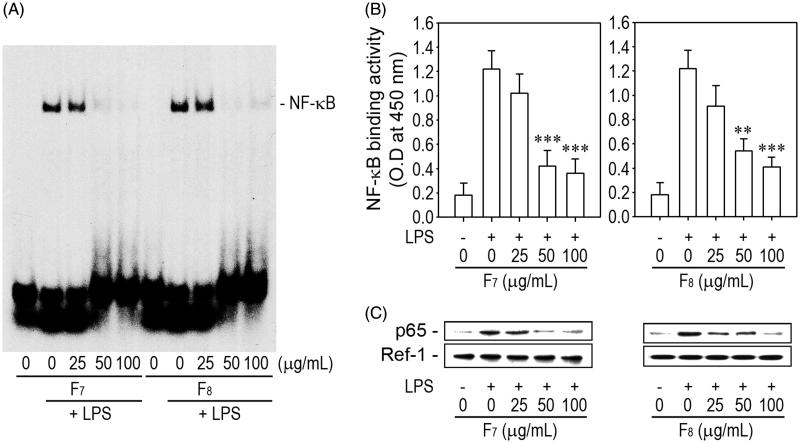
The IRG active fractions inhibit NF-κB DNA-binding in LPS-stimulated macrophages. Cells were pretreated with the indicated concentrations (0–100 μg/mL) of F_7_ or F_8_ for 1 h just prior to stimulation with 1 μg/mL LPS. After 1 h of stimulation, nuclear fractions were prepared from the cells and analyzed for NF-κB binding activity by (A) EMSA, (B) nonisotopic enzymatic assay and (C) Western blot analysis. EMSA and immunoblotting results are representative of three different experiments. **p < 0.05 and ***p < 0.01 vs. LPS treatment alone.

As NF-κB regulates the signal transduction pathways involved in inflammation, we performed EMSA to examine the effects of F_7_ and F_8_ on NF-κB DNA-binding in macrophages stimulated with 1 μg/mL LPS. As shown in Figure 6(A), LPS treatment increased the binding of NF-κB to its specific DNA sequence and this increase was almost completely inhibited by pretreatment with 50 μg/mL of F_7_ or F_8_. Enzymatic assay also revealed that the addition of F_7_ or F_8_ (more than 25 μg/mL) significantly suppressed LPS-induced p65-binding activity (Figure 6(B)). In support of this result, LPS-mediated increase in the nuclear p65 level was diminished by combined treatment with each of the active fractions (Figure 6(C)).

### Protection of septic death by the IRG active fractions

**Figure 7.**
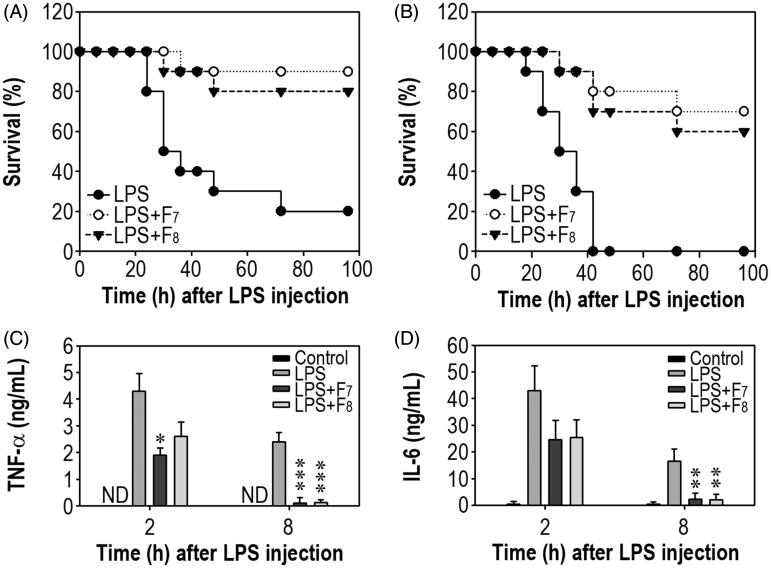
Oral treatment with the IRG active fractions protects mice against septic damage. (A) Female BALB/c and (B) ICR mice were injected with LPS (30 mg/kg body weight) one day after the last administration of the fractions. Thereafter, the survival of mice in the sepsis group was monitored every 6 h for 4 days. Serum samples were collected from BALB/c mice that had been orally administered each fraction at 2 and 8 h after the LPS injections. The levels of serum (C) TNF-α and (D) IL-6 were then determined. *p < 0.05, **p < 0.01 and ***p < 0.001 vs. mice injected with LPS alone. ND: not detected.

We further examined the ability of F_7_ and F_8_ to protect mice against LPS-mediated septic damage (Figure (7)). The survival rate of BALB/c mice injected intraperitoneally with LPS gradually decreased over time. Specifically, LPS-injected mice showed 80, 50, 40, 30 and 20% survival at 24, 30, 36, 48 and 72 h, respectively, after the LPS injection (Figure 7(A)). In contrast, oral administration of either F_7_ or F_8_ improved the survival rates to 90 and 80%, respectively, even at 96 h after the LPS injection. While the survival rate of ICR mice was 0% at 42 h after the LPS injection, administration of F_7_ or F_8_ improved the survival rates to 70 and 60%, respectively, at 72 h after LPS treatment (Figure 7(B)). Since the pathogenic mechanism of sepsis involves intravascular inflammation mediated by various proinflammatory cytokines, we measured the serum levels of TNF-α and IL-6 in BALB/c mice. Serum TNF-α level in the untreated control mice was undetectable, whereas TNF-α level in the LPS-treated mice rapidly increased up to 4.3 ± 0.67 ng/mL at 2 h and decreased to 2.4 ± 0.34 ng/mL at 8 h after the LPS injection (Figure 7(C)). Oral treatment with F_7_, but not F_8_, significantly (*p* < 0.05) prevented the acute increase in serum TNF-α at 2 h post-LPS injection. However, oral administration with F_7_ or F_8_ decreased significantly (*p* < 0.001) the serum TNF-α levels at 8 h post-LPS injection. The serum IL-6 concentrations were also dramatically increased in the LPS-treated mice compared with the untreated control mice (Figure 7(D)). In contrast, both F_7_ and F_8_ almost completely reduced (*p* < 0.01) the serum IL-6 levels at 8 h after the LPS injection. When the serum TNF-α and IL-6 levels in LPS-injected ICR mice were determined, the F_7_ and F_8_ also showed inhibitory effects on the proinflammatory cytokines (data not shown).

## Discussion

In contrast to Fe^3+^-dependent hydroxyl radical scavenging assays that indirectly measure the radical scavenging activity, the DPPH assay is a direct approach for evaluating free radical scavenging potential. DPPH radical is a stable radical that is widely used to estimate the antioxidant activity of plant and microbial extracts (Hu & Kitts 2000; Jiménez-Suárez et al. 2016). However, this radical is not formed within living organisms; rather superoxide anions are the most common free radicals *in vivo*. These free radicals are generated in a variety of biological systems, either by auto-oxidation or by enzymes (von Harsdorf et al. 1999). It is known that the concentration of superoxide anions can be increased under the conditions of oxidative stress and other related situations (Mates & Sanchez-Jimenez 2000). In addition, superoxide anions produce other additional damaging free radicals and oxidizing agents (Lee et al. 2012). Thus, the ability to scavenge superoxide anions is important for preventing oxidative damage to cells and tissues. Furthermore, the scavenging activities of IRG active fractions on hydroxyl radicals and superoxide anions were higher than that of the extracts from cactus (Lee et al. 2002) and *Rhus verniciflua* Stokes (Anacardiaceae) (Lee et al. 2002). Accordingly, our data indicate that the IRG active fractions are potential scavengers of superoxide anions and hydroxyl radicals.

Inflammation is a host response to endotoxins that is mediated by complex processes and characterized by several classic signs. The increases in the levels of NO and proinflammatory cytokines are the common phenomenon in the processes of inflammatory responses (Joubert & Malan 2011). NO is produced in macrophages after the enzymatic activation of iNOS (Kobayashi 2010). Overproduction of NO, TNF-α and IL-6 by macrophages leads to various pathological disorders such as carcinogenicity, cytotoxicity and autoimmune diseases (Kobayashi 2010; Joubert & Malan 2011). Therefore, achieving efficient inhibition on NO and proinflammatory cytokines is regarded as an attractive approach by which a variety of disorders triggered by inflammatory mediators can be alleviated.

It has been demonstrated that bioactive substances having antioxidant potential exert anti-inflammatory properties (Ferretti et al. 2010). The current findings support a close relation between the free radical scavenging potential and the activity to suppress macrophage hyperactivation and to decrease the levels of inflammatory mediators. Many studies have shown that phenolic compounds are able to scavenge reactive oxidants as well as to inhibit inflammatory responses. It was also known that plants contain abundant phenolic acids and polyphenolic compounds which show pharmacological and medicinal activities (Cuevas Montilla et al. 2011; Kamiyama & Shibamoto 2012; Tunin et al. 2016). Similarly, our results highlight that the two active fractions, F_7_ and F_8_, have greater antioxidant and anti-inflammatory potentials than other fractions and consist primarily of phenolic acids and flavonoid compounds. HPLC analysis revealed that the active fractions contained caffeic acid, ferulic acid, myricetin and kaempferol as the main active components. These results strongly support that phenolic acid derivatives and flavonoids are the active components responsible for the antioxidant and anti-inflammatory activities of IRG chloroform-soluble extract. The present findings also suggest that the mixtures with phenolic acids and flavonoids may inhibit LPS-stimulated inflammatory responses more efficiently and synergistically than did a single phenolic or flavonoidic compound. This was because the active fractions prevented LPS-stimulated production of TNF-α in macrophages more greatly than did myricetin or kaempferol at the same concentration.

NF-κB is a critical factor that regulates the signal transduction pathways involved in LPS-induced inflammation. Exposure to endotoxins stimulates iNOS induction and leads to the subsequent production of NO and proinflammatory cytokines through the activation of NF-κB (Doyle & O'Neill 2006). Accumulated findings also demonstrate that the potential of active substances to inhibit the LPS-stimulated hyperactivation of macrophages is related to their ability to suppress NF-κB-mediated pathways (Wu et al. 2004; Shin et al. 2009). Regarding the mechanisms by the IRG active fractions inhibit the LPS-mediated induction of NO, TNF-α, iNOS and COX-2, the current findings suggest the involvement of NF-κB-mediated signalling. Nonisotopic enzymatic and Western blot assays also indicate that p65 is the primary subunit that dimerizes with p50 or p52 and mediates DNA binding of NF-κB. It is important to note that NF-κB activation is tightly regulated by MAPKs. ERK and p38 MAPK are the primary mediators of the signalling pathways involved in macrophage activation in response to microbial pathogens (Gaestel et al. 2007; Li et al. 2016). In combination with previous findings, our results imply that the active fractions F_7_ and F_8_ suppress inflammatory responses by inhibiting ERK- and/or p38 MAPK-NF-κB-mediated pathways.

While chronic inflammation is related to various degenerative diseases, acute inflammation can cause death directly. The pathogenic mechanism of sepsis primarily involves intravascular inflammation mediated by various cytokines and chemokines (Patil et al. 2016). Therefore, the mechanisms by which an active material protects against acute and severe inflammation are often determined by measuring the serum levels of proinflammatory cytokines in a suitable animal model of sepsis. In general, serum TNF-α is barely detectable in untreated control BALB/c mice, while IL-6 level ranges from 1 to 2 ng/mL (Blanque et al. 1996). The latter finding is consistent with the present study, in which IL-6 was only slightly detectable. Following LPS stimulation, serum TNF-α levels are known to exhibit a rapid and significant increase, as do the IL-6 levels (Kotanidou et al. 2002; Cheng et al. 2016). Although specific increases in inflammatory cytokines are known to differ according to the LPS dose and/or the animal model used, most studies have supported negative correlations between survival rate and the serum levels of these inflammatory cytokines (Blanque et al. 1996; Kotanidou et al. 2002; Wang et al. 2015). This observation indicates that the potential of an active material to reduce the serum levels of inflammatory cytokines is directly related to its ability to block LPS-mediated acute inflammation. Consequently, a hypothesis that diets with anti-inflammatory substances may improve host immunity could be supported by our findings that the IRG active fractions protect mice against septic damage by decreasing the serum levels of inflammatory cytokines.

## Conclusions

This study demonstrates that IRG active fractions have antioxidant and anti-inflammatory effects *in vitro* and *in vivo*. The primary mechanisms by which these active fractions exert their anti-inflammatory activities involve the suppression of NF-κB DNA-binding activation via the inhibition of ERK and p38 MAPK phosphorylation. Overall, our results indicate that phenolic acids and flavonoid compounds are the primary bioactive constituents of the IRG active fractions. Taken as a whole, we suggest that IRG silage is useful as a diet additive for human as well as monogastric animals.

## Supplementary Material

Jeong-Chae__Lee_et_al_supplemental_content.zip
