# Supplementary material for: Antioxidant, anti-inflammatory and anti-septic potential of phenolic acids and flavonoid fractions isolated from Lolium multiflorum
Source: Pharm Biol. 2016 Dec 9;55(1):611–9. doi: 10.1080/13880209.2016.1266673 (PMC6130696; doi:10.1080/13880209.2016.1266673)
Supplement: Jeong-Chae__Lee_et_al_supplemental_content.zip [file IPHB_A_1266673_SM4181.zip › Jeong-Chae Lee et al supplemental content.pdf]

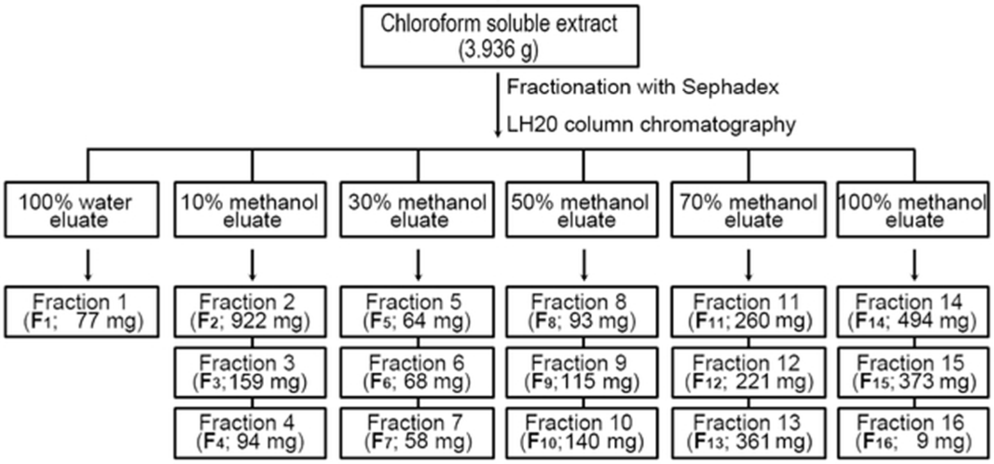

53x25mm (300 x 300 DPI)

For Review Only

Peak No.	Compound	Chemical structure	Retention time (min)
1	(+)-Catechin	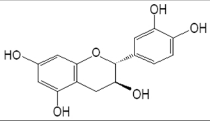	9.675
2	Caffeic acid	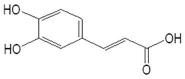	10.517
3	Ferulic acid	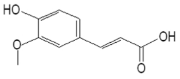	11.608
4	p-Coumaric acid	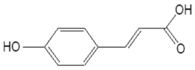	11.775
5	Syringic aldehyde	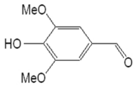	12.133
6	Myricetin	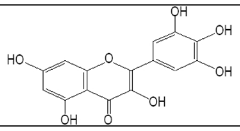	12.775
7	Propyl gallate	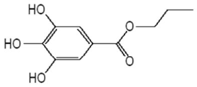	13.058
8	Quercetin	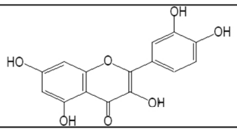	14.008
9	Kampferol	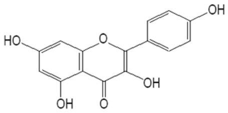	15.217

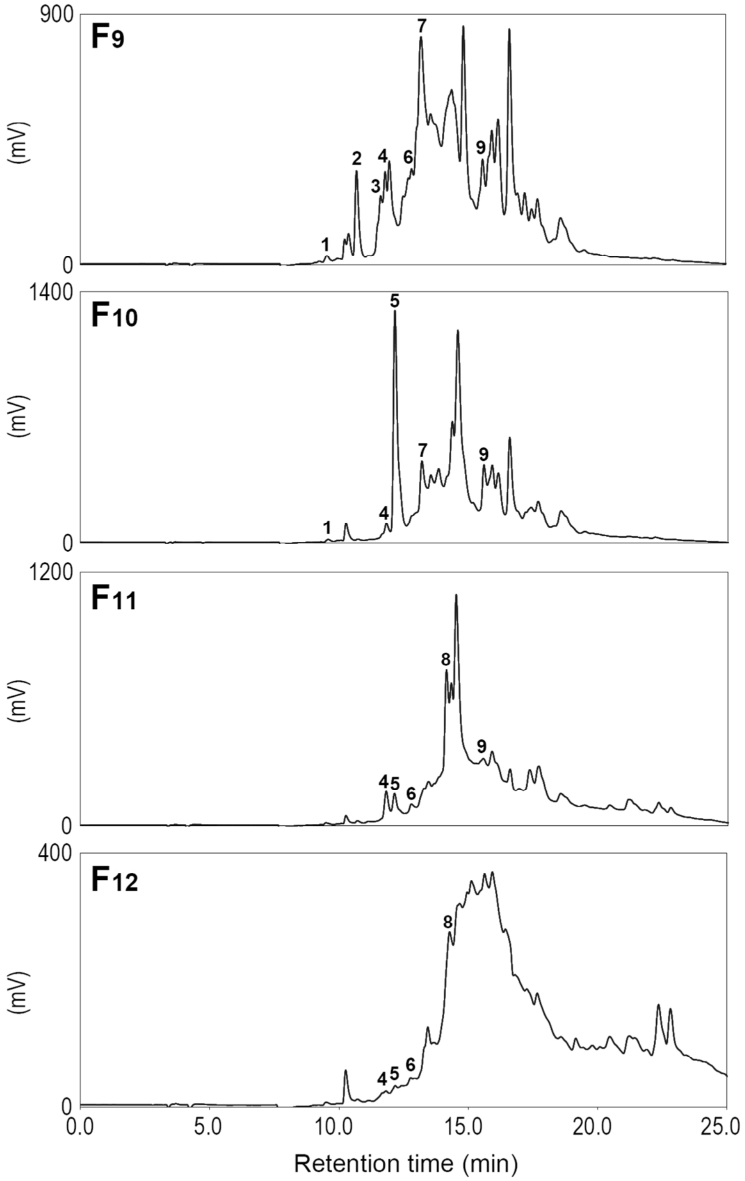

135x211mm (300 x 300 DPI)

Supplemental Figure legends

Supplement Fig. 1. Scheme for preparation of the active fractions from the chloroform-soluble extract of the IRG methanol extract.

Supplement Fig. 2. Chemical structures and retention times of authentic compounds.

Supplement Fig. 3. HPLC chromatograms of the fractions F_9, F_{10}, F_{11}, and F_{12}.

For Peer Review Only
